# The Incompetence of Mosquitoes—Can Zika Virus Be Adapted To Infect *Culex tarsalis* Cells?

**DOI:** 10.1128/msphere.00015-23

**Published:** 2023-02-16

**Authors:** Emily N. Gallichotte, Demetrios Samaras, Reyes A. Murrieta, Nicole R. Sexton, Alexis Robison, Michael C. Young, Alex D. Byas, Gregory D. Ebel, Claudia Rückert

**Affiliations:** a Center for Vector-Borne Infectious Diseases, Department of Microbiology, Immunology, and Pathology, Colorado State University, Fort Collins, Colorado, USA; b Department of Biochemistry and Molecular Biology, College of Agriculture, Biotechnology & Natural Resources, University of Nevada, Reno, Nevada, USA; Stanford University School of Medicine

**Keywords:** arbovirus, mosquito, species specificity, virology

## Abstract

The molecular evolutionary mechanisms underpinning virus-host interactions are increasingly recognized as key drivers of virus emergence, host specificity, and the likelihood that viruses can undergo a host shift that alters epidemiology and transmission biology. Zika virus (ZIKV) is mainly transmitted between humans by Aedes aegypti mosquitoes. However, the 2015 to 2017 outbreak stimulated discussion regarding the role of *Culex* spp. mosquitoes in transmission. Reports of ZIKV-infected *Culex* mosquitoes, in nature and under laboratory conditions, resulted in public and scientific confusion. We previously found that Puerto Rican ZIKV does not infect colonized Culex quinquefasciatus, Culex pipiens, or *Culex tarsalis*, but some studies suggest they may be competent ZIKV vectors. Therefore, we attempted to adapt ZIKV to *Cx. tarsalis* by serially passaging virus on cocultured *Ae. aegypti* (Aag2) and *Cx. tarsalis* (CT) cells to identify viral determinants of species specificity. Increasing fractions of CT cells resulted in decreased overall virus titer and no enhancement of *Culex* cell or mosquito infection. Next-generation sequencing of cocultured virus passages revealed synonymous and nonsynonymous variants throughout the genome that arose as CT cell fractions increased. We generated nine recombinant ZIKVs containing combinations of the variants of interest. None of these viruses showed increased infection of *Culex* cells or mosquitoes, demonstrating that variants associated with passaging were not specific to increased *Culex* infection. These results reveal the challenge of a virus adapting to a new host, even when pushed to adapt artificially. Importantly, they also demonstrate that while ZIKV may occasionally infect *Culex* mosquitoes, *Aedes* mosquitoes likely drive transmission and human risk.

**IMPORTANCE** ZIKV is mainly transmitted between humans by *Aedes* mosquitoes. In nature, ZIKV-infected *Culex* mosquitoes have been found, and ZIKV infrequently infects *Culex* mosquitoes under laboratory conditions. Yet, most studies show that *Culex* mosquitoes are not competent vectors for ZIKV. We attempted to adapt ZIKV to *Culex* cells to identify viral determinants of species specificity. We sequenced ZIKV after it was passaged on a mixture of *Aedes* and *Culex* cells and found that it acquired many variants. We generated recombinant viruses containing combinations of the variants of interest to determine if any of these changes enhance infection in *Culex* cells or mosquitoes. Recombinant viruses did not show increased infection in *Culex* cells or mosquitoes, but some variants increased infection in *Aedes* cells, suggesting adaptation to those cells instead. These results reveal that arbovirus species specificity is complex, and that virus adaptation to a new genus of mosquito vectors likely requires multiple genetic changes.

## INTRODUCTION

Zika virus (ZIKV; genus *Flavivirus*) is an arthropod-borne virus (arbovirus) transmitted mainly by Aedes aegypti and secondarily by *Ae. albopictus* mosquitoes across large parts of the world. ZIKV was first isolated from a sentinel rhesus monkey in the Ziika forest of Uganda in 1947 (1) and only a few sporadic cases in various African countries were reported over the next 60 years. In 2007 however, a large outbreak was reported on Yap Island in Indonesia, infecting over 70% of the population (2) and a second Asian lineage of ZIKV was identified in 2012 (3). During 2013 and 2014, the virus caused outbreaks in various Pacific islands, such as French Polynesia, Easter Island, the Cook Islands, and New Caledonia (4, 5), and was likely introduced to the Americas in late 2013 or early 2014 (6), but remained undetected until early 2015. The mosquito vectors for these outbreaks were thought to be *Ae. aegypti* and *Ae. polynesiensis* (specifically in the Pacific islands) (7, 8). However, once ZIKV reached Brazil, researchers increasingly started implicating the Southern house mosquito, Culex quinquefasciatus, in ZIKV transmission (7, 9).

Many studies have evaluated the ability of ZIKV to infect and be transmitted by various *Culex* spp. mosquitoes. ZIKV has been isolated from pools of *Cx. quinquefasciatus* collected during the pandemic, but generally with low levels of viral RNA present (10, 11). There are two laboratory studies demonstrating substantial infection rates (+80%) of *Cx. quinquefasciatus* mosquitoes, and in one study, 80% transmission (12, 13). Conversely, there are over 17 studies showing none to minimal infection of, and no transmission of ZIKV by *Cx. quinquefasciatus* mosquitoes (9, 11, 14–18). Studies evaluating additional *Culex* species, including *Cx. tarsalis*, *Cx. pipiens*, *Cx. restuans*, and *Cx. coronator*, reveal similar results; while *Culex* mosquitoes can occasionally become infected, there is no convincing evidence that they are competent vectors for ZIKV (19), particularly compared with established *Aedes* vectors. The literature on the subject remains controversial, and there are at least three potential explanations for this variability: successful ZIKV infection of *Culex* mosquitoes depends on (i) the specific ZIKV genotype, (ii) the genetic background of the mosquito population, or (iii) complex environmental factors (e.g., microbiome).

There are many instances of arboviruses acquiring small genetic changes that lead to either improved fitness in mosquitoes or the ability to infect a new species more efficiently. Chikungunya virus, which is most frequently transmitted by *Ae. aegypti* mosquitoes, acquired a single coding change in the envelope protein, which led to a significant increase in the virus's ability to be transmitted by *Ae. albopictus* mosquitoes (20). As West Nile virus spread throughout North America, a new subtype (WN02) emerged containing a single amino acid change (E-V159A) (21, 22). This change led to increased replication in *Cx. pipiens* and *Cx. tarsalis* mosquitoes and a shorter extrinsic incubation period (22, 23), leading to the displacement of the original NY99 genotype. In a laboratory setting, Mayaro virus was adapted to *Ae. aegypti* cells, which identified a single envelope coding change (E2-T179N) that resulted in increased transmission by *Ae. aegypti* mosquitoes (24). In a similar experiment, Sindbis virus was passaged on cocultures of BHK-21 (highly susceptible) and CHO cells (poorly susceptible) to improve fitness on CHO cells. The authors showed that a gradual increase in the “novel host cell” (CHO) over 25 passages resulted in increased Sindbis virus fitness in CHO cells with the virus acquiring many genetic changes likely contributing to the improved fitness (25).

In this study, we tested vector competence of multiple *Culex* spp. mosquitoes to three ZIKV isolates, both through artificial bloodmeal and intrathoracic microinjection to set a baseline of vector competence for our existing colonies. We then aimed to determine whether the Puerto Rican ZIKV isolate can be adapted to replication in *Cx. tarsalis* CT cells through a coculture approach using highly susceptible *Ae. aegypti* Aag2 and less susceptible CT cells, similar to previous experiments by Morley et al. (25). While this is an artificial set-up and not meant to directly mimic natural conditions of ZIKV replication, it creates conditions where CT cells are constantly exposed to ZIKV, possibly allowing for the selection of adaptive mutations to arise. For our purposes, Aag2 and CT cells are highly suitable for this type of approach as they grow under the same culture conditions. We infected a culture with predominantly Aag2 cells and passaged the virus through cocultures with ever increasing proportions of CT cells (see results section). Once we reached 90% CT cells within the coculture (18 passages), a point where we may have “adapted” the virus to growth on CT cells, we sequenced virus populations from all coculture passages to identify single nucleotide variants (SNVs) of interest and associated with increasing ratios of CT cells. Using our infectious clone, we generated recombinant ZIKVs containing the SNVs of interest and evaluated viruses for ability to infect *Aedes* and *Culex* cells and mosquitoes. We found that while the virus accumulated many coding and noncoding changes during passaging, none of the variants studied improved infection of *Culex* cells or mosquitoes. These results reveal that likely multiple genetic changes would need to occur for ZIKV to be efficiently transmitted by *Culex* in nature.

## RESULTS

### Infection of *Cx. quinquefasciatus* with three strains of ZIKV.

We first sought to determine if *Culex* mosquitoes are competent vectors of ZIKV using three genetically distinct virus strains. *Cx. quinquefasciatus* mosquitoes were provided an infectious virus bloodmeal or injected intrathoracically with virus and held at either 28°C or 32°C postinfection for 7 days (Table 1). We observed low infection rates (<2%) following bloodmeal infection, independent of the virus strain or external incubation temperature (*P* > 0.05). Due to these extremely low infection rates, we did not test for dissemination or transmission. Following intrathoracic injection, which bypasses the midgut barrier, we observed significantly higher infection rates for the Ugandan strain (MR766; 20%) and the Dakar strain (41525; 57%), but there was still no dissemination to the saliva. These data confirm that while *Culex* mosquitoes can occasionally become infected with ZIKV (26, 27), there are likely multiple barriers to infection and transmission. Further, they confirm virus strain-dependent variation in replication within mosquitoes.

**TABLE 1:** Vector competence of *Cx. quinquefasciatus* for three ZIKV strains^*a*^

Infection by	Extrinsic incubation temp	ZIKV strain	ZIKV pos bodies	ZIKV pos legs/wings	ZIKV pos saliva
Bloodmeal	28°C	PRVABC59	1/72 (1.4%)	Not tested	Not tested
		MR766	1/66 (1.5%)	Not tested	Not tested
		41525	1/72 (1.4%)	Not tested	Not tested
Bloodmeal	32°C	PRVABC59	1/108 (0.9%)	Not tested	Not tested
		MR766	2/108 (1.9%)	Not tested	Not tested
		41525	2/108 (1.9%)	Not tested	Not tested
Intrathoracic injection	28°C	PRVABC59	0/30 (0%)	0/30 (0%)	0/30 (0%)
		MR766	6/30 (20%)^*b*^	1/30 (3.3%)	0/30 (0%)
		41525	17/30 (57%)^*c*^	10/30 (33%)^*d*^	0/30 (0%)

a ZIKV infection was determined by standard plaque assay on Vero cells. Significant differences between MR766 and 41525 compared to PRVABC59 for each tissue type and infection condition using a Chi-square test are shown.

b *P* < 0.005.

c *P* < 0.0005.

d *P* < 0.0001.

### Low level infection of *Culex* cells with ZIKV.

**FIG 1:**
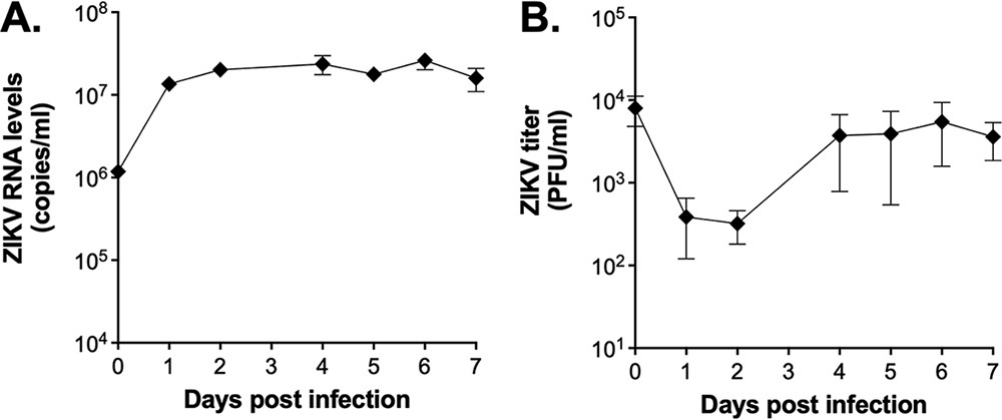
Low level replication of ZIKV in CT cells. *Cx. tarsalis* CT cells were infected at an MOI of 20 with ZIKV PRVABC59 virus, supernatant collected daily, and analyzed for (A) viral RNA via qRT-PCR and (B) infectious virus via plaque assays. Experiment was performed in biological triplicate (mean ± standard deviation).

We next wanted to see if a *Cx. tarsalis* cell line (CT cells, originally derived from embryonic tissue) could support low level ZIKV replication, since it has previously been shown that *Cx. tarsalis* mosquitoes are more easily infected than other *Culex* spp. (27). We infected cells at a high multiplicity of infection (MOI = 20), sampled supernatant daily, and assayed for both viral RNA and infectious virus (Fig. 1). We observed an increase in extracellular viral RNA in the first 2 days to a level that was sustained throughout the time course (Fig. 1A). While infectious virus levels dropped early following infection, levels increased by 4 days postinfection and remained elevated, suggesting some production of infectious ZIKV by CT cells (Fig. 1B).

### Adapting ZIKV to *Culex* cells.

**FIG 2:**
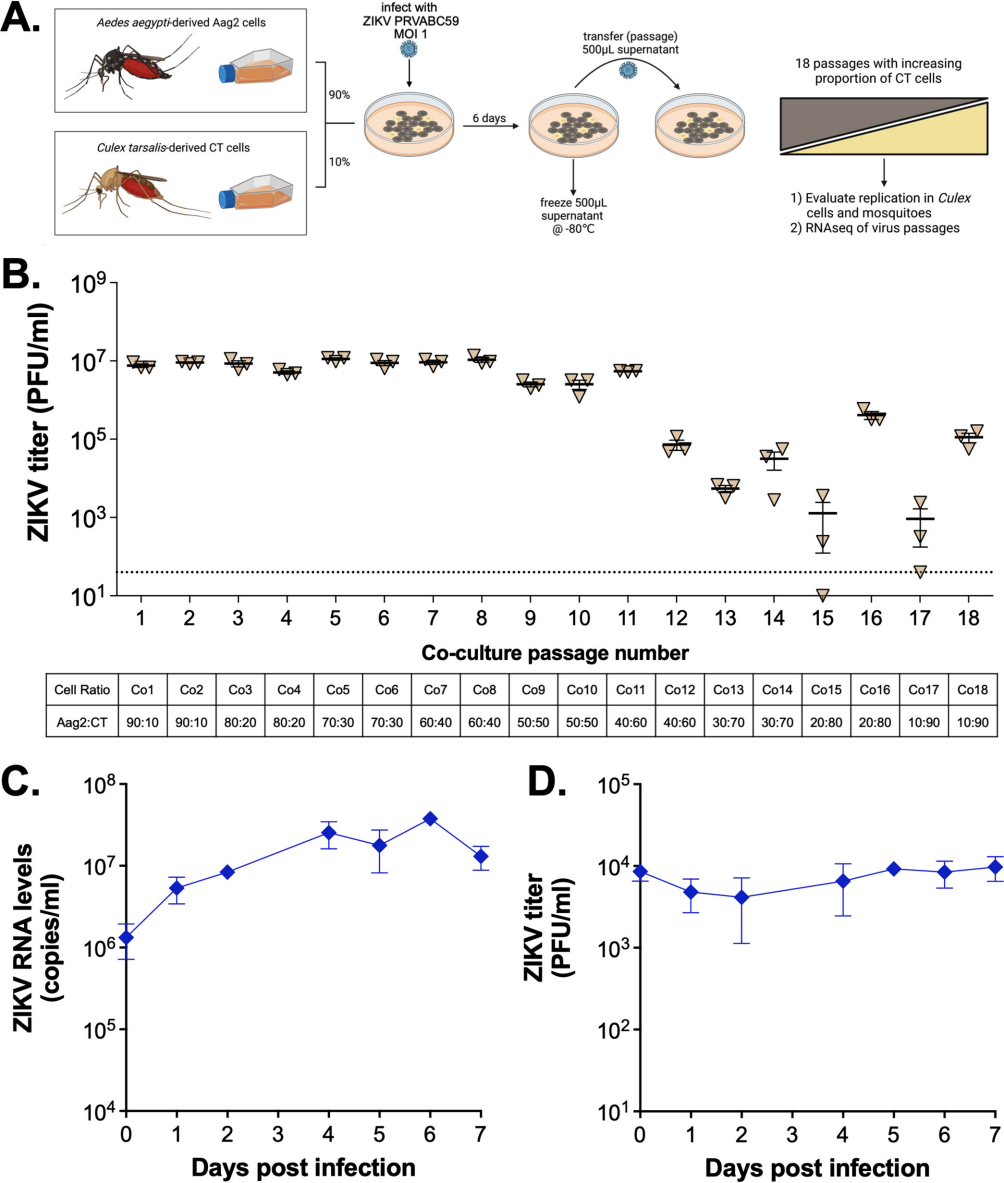
ZIKV passaging on Aag2 and CT cells. (A) Schematic of passaging experimental design. *Ae. aegypti*-derived Aag2 and *Cx. tarsalis*-derived CT cells were mixed at an initial ratio of 90:10, infected with ZIKV PRVABC59 isolate for 6 days, then culture supernatant was passaged onto new cells. The ratio of Aag2:CT slowly decreased to a final 10:90 ratio over the course of 18 passages. Passaging experiment was performed in biological triplicate. (B) After each passage, supernatant was assayed for infectious virus. (C–D) *Cx. tarsalis* CT cells were infected at an MOI of 20 with each triplicate of ZIKV passage 18 coculture virus that was passaged once on Vero cells (Co18.1V, Co18.2V and Co18.3V), supernatant collected daily, and analyzed for (C) viral RNA via qRT-PCR and (D) infectious virus via plaque assays. Growth curve experiment was performed in biological triplicate (mean ± standard deviation). Dashed line represents limit of detection. Graphics were generated using BioRender.com.

Because we saw low level infection of ZIKV in both *Culex* mosquitoes and cells, we attempted to adapt ZIKV to *Culex* cells through copassaging with a highly susceptible cell line to further increase infection and replication efficiency (Fig. 2). Aag2 cells (highly competent for ZIKV) were mixed with CT cells, infected with ZIKV for 6 days, then supernatant was passaged onto new cocultured cells. The ratio of Aag2 to CT cells started high (90% Aag2 to 10% CT), and gradually decreased with each passage until CT cells were 90% by the final passage (Fig. 2A). Virus titers began to decrease when CT cells were >60% of the population, but infectious virus was still detected even in the last passage (Fig. 2B). Due to the low level of infectious virus in the last passage, this passage was expanded once on Vero cells (Co18.1-3V) and evaluated for its ability to infect CT cells at an MOI of 20 (Fig. 2C and D). We saw low-level infection of the passaged virus on CT cells (Fig. 2C and D), similar to the original ZIKV PRVABC59 (Fig. 1).

### Infection of three *Culex* species with passaged virus stocks.

We next evaluated the ability of the passaged virus to infect three species of *Culex* mosquitoes compared to the parental WT ZIKV via an infectious bloodmeal (Table 2). Due to the low titer of p18 virus (Fig. 2B), we first grew a high titer stock on Vero cells (Co18.1-3V) and exposed *Cx. tarsalis*, *Cx. pipiens*, and *Cx. quinquefasciatus* mosquitoes with these stocks (1 × 10^7^ PFU/mL). We found low levels of ZIKV infection across all species, with *Cx. tarsalis* having the highest levels of infection. The three cocultured virus stocks showed variable low levels of infection in *Cx. tarsalis*, ranging from 2.3 to 8.3% positive (compared to 3.3% positive with WT ZIKV) (Table 2). Despite these minor fluctuations, there were no significant differences between infection rates (*P* > 0.05).

**TABLE 2:** Vector competence of *Culex* mosquitoes with passage 18 virus stocks^*a*^

Species	WT ZIKV	Co18.1V	Co18.2V	Co18.3V
*Cx. tarsalis*	2/60 (3.3%)	5/60 (8.3%)	2/60 (3.3%)	1/44 (2.3%)
*Cx. pipiens*	0/32 (0%)	1/40 (2.5%)	0/37 (0%)	0/32 (0%)
*Cx. quinquefasciatus*	0/47 (0%)	0/40 (0%)	0/40 (0%)	0/32 (0%)

a ZIKV infection was determined by standard plaque assay on Vero cells. Co18.1-3V were not significantly different than WT ZIKV for any mosquito species by a Chi-square test (*P* > 0.05).

### Next-generation-sequencing of ZIKV passages.

**FIG 3:**
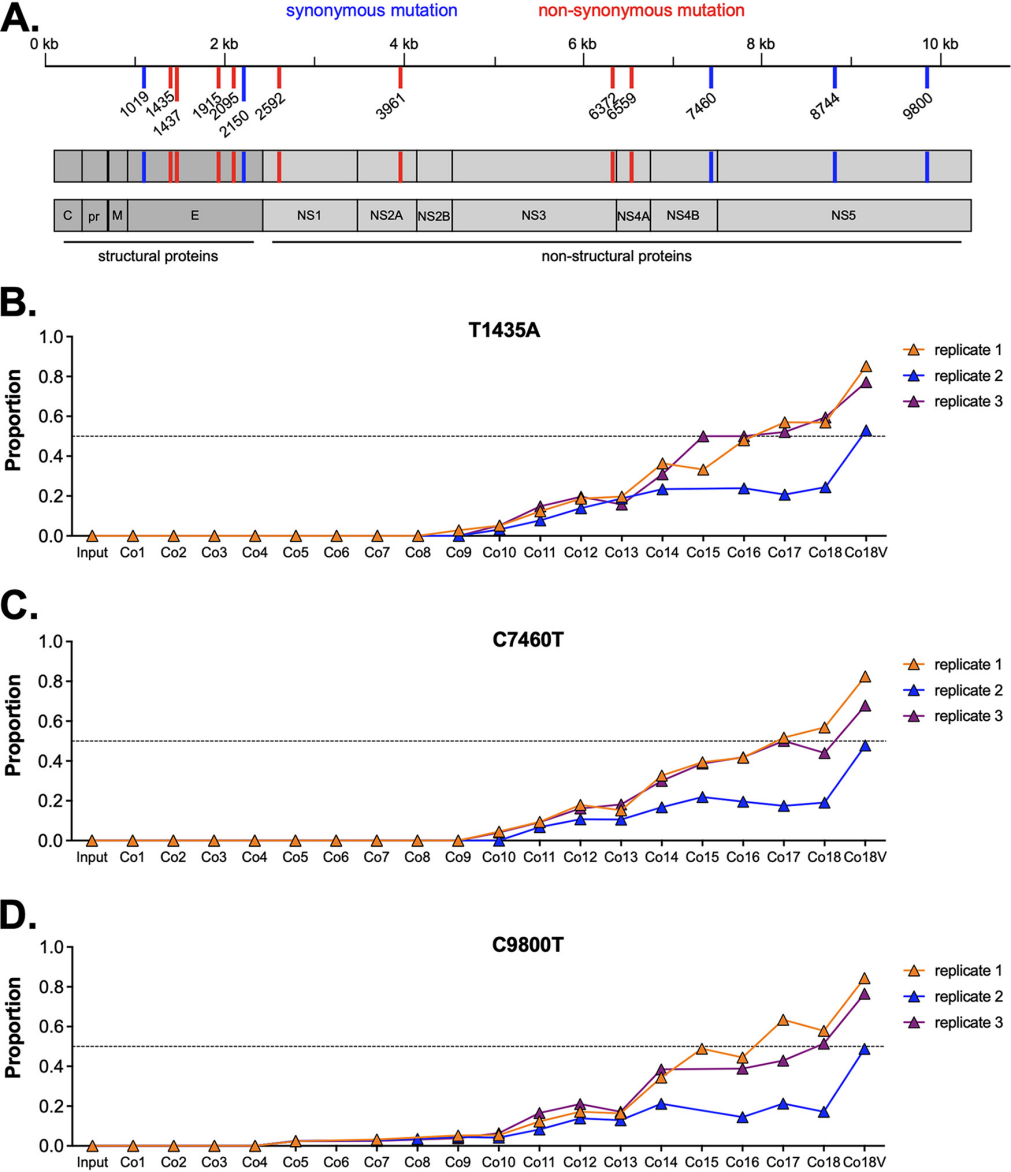
SNVs associated with ZIKV passaging on Aag2/CT cocultures. (A) Schematic overview of SNVs of interest along the ZIKV PRVABC59 genome. (B–D) Proportion of SNVs out of total virus population (i.e., variant frequencies) for three mutations of interest (B) T1435A, (C) C7460T, (D) and C9800T. Variant frequencies for each passage and each independent passaging replicate are shown. Co18V indicates the Vero propagated virus stock generated by inoculation of Vero cells with Co18 culture supernatant. Dashed line represents 0.5.

To determine genetic changes resulting from passaging of ZIKV on cocultured Aag2 and CT cells, we performed total RNA sequencing of the cocultured stocks after each passage (Co1-18) and from the Vero propagated high titer Co18 stocks (Co18.1-3V). We found 13 single SNVs of interest (Table 3, Fig. 3A), as determined by their increasing frequency once CT cell ratios reached and surpassed 50% of the coculture. We hypothesized that these mutations arose in frequency because of the increasing proportion of CT cells in the culture. Numerous other SNVs were detected that either did not change in frequency, were reduced in frequency over coculture passaging, or changed in frequency randomly between passages. The 13 SNVs of interest spanned the entire genome, were comprised of both synonymous (non-coding) and nonsynonymous (coding) changes and were equally distributed among structural and nonstructural proteins (Fig. 3A). Three such mutations (T1435A, C7460T, C9800T) were found in all three replicate passages and became part of the consensus sequence (>50% allele frequency) by passage 18 in two out of the three replicates (Fig. 3B to D). Frequencies for these mutations were near identical between replicates, suggesting that these SNVs were present on the same ZIKV haplotype. Other SNVs increased to a lower extent, such as A1437G, which gradually increased to ~20% at passage 16, but then decreased in the following passage. The SNVs T1435A and A1437G were of particular interest due to their location at (1437) or immediately adjacent to (1435) the glycosylation site in E (N154). Both SNVs rose in frequency over passaging and resulted in a negatively charged amino acid with a probable (V153D) and definite (N154D) loss of glycosylation at this site.

**TABLE 3:** Summary of SNVs of interest

Genome position	nt constant	nt variant	Viral protein	aa position	S/NS	aa change	Stock SNV frequency	Co18.1-3 SNV frequency^*a*^	Detected in replicate
1019	T	C	E	14	S	-	<0.01	0.08	Co18.3
1435	T	A	E	153	NS	V > D	<0.01	0.53	Co18.1-3
1437	A	G	E	154	NS	N > D	<0.01	0.14	Co18.1-3
1915	C	T	E	313	NS	T > I	<0.01	0.20	Co18.2
2095	A	G	E	373	NS	K > R	<0.01	0.06	Co18.2
2150	C	T	E	391	S	-	<0.01	0.06	Co18.2
2592	C	T	NS1	35	NS	H > Y	<0.01	0.28	Co18.1-2
3961	T	C	NS2A	139	NS	I > T	<0.01	0.06	Co18.2-3
6372	A	C	NS3	590	NS	K > Q	<0.01	0.19	Co18.1-3
6559	T	C	NS4A	32	NS	M > T	<0.01	0.10	Co18.1
7460	C	T	NS4B	182	S	-	<0.01	0.39	Co18.1-3
8744	A	G	NS5	359	S	-	<0.01	0.24	Co18.1-2
9800	C	T	NS5	711	S	-	<0.01	0.42	Co18.1-3

a Whenever an SNV was represented in more than one replicate, the mean SNV frequency is shown.

### Generating viral mutants incorporating SNVs.

**FIG 4:**
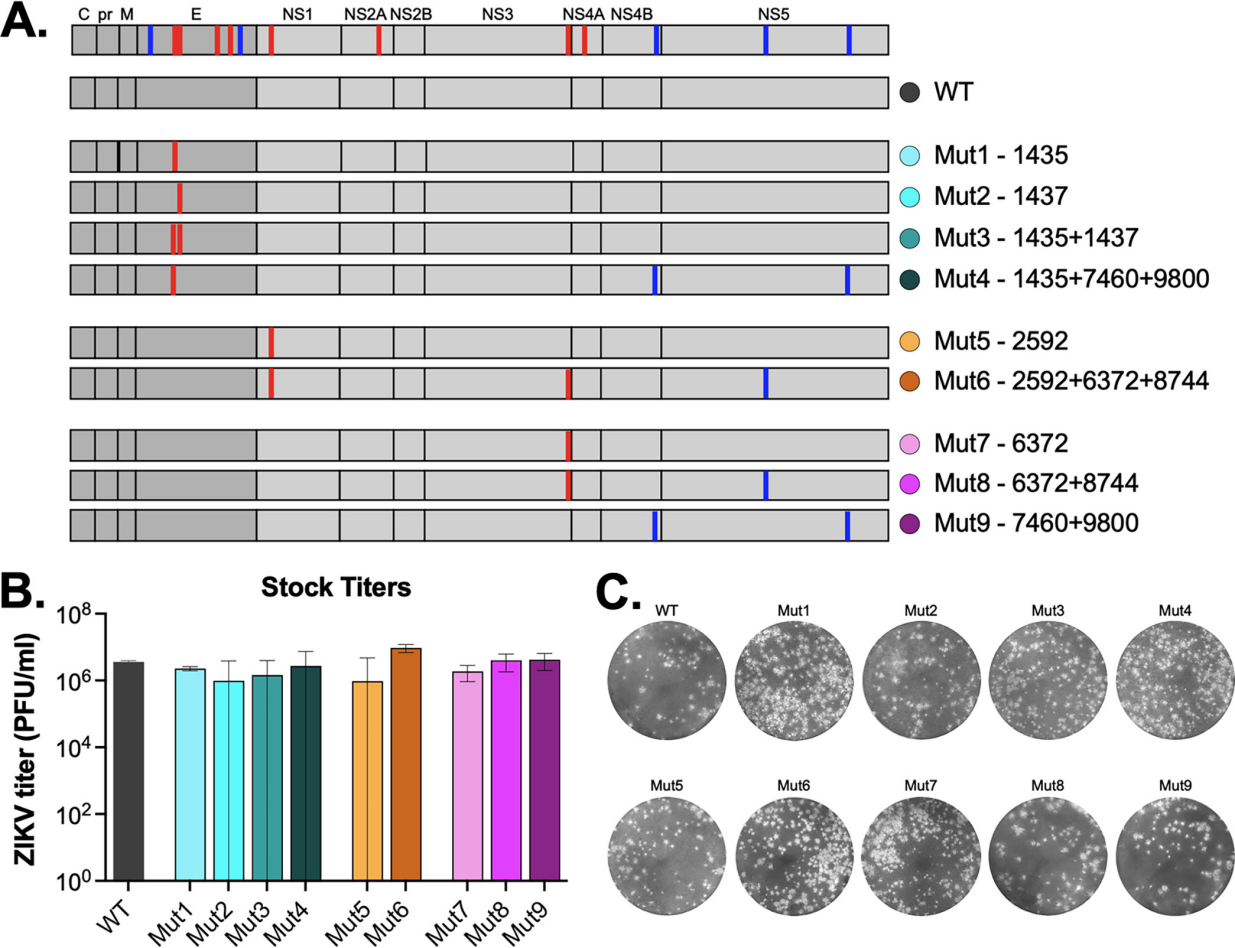
Design and characterization of ZIKV recombinant viruses. (A) Schematic of ZIKV genome, with synonymous (blue) and nonsynonymous (red) variants of interest shown as lines. Genome location and type (synonymous versus nonsynonymous) of variants of each of the recombinant viruses is shown (Mut1-9). (B) Recombinant viruses were recovered, stocks generated, and assayed for infectious virus titer (performed in technical duplicate, mean ± 95% confidence intervals). (C) Plaque morphology of ZIKV recombinant viruses on Vero cells.

To determine the role of any of the SNVs either individually, or in combination with others, we generated mutants using our ZIKV PRVABC59 reverse genetics infectious clone (28) (Fig. 4A). Recombinant viruses were designed for those SNVs considered of highest interest: T1435A (Mut1) and A1437G (Mut2), due to their location on E. A combination of both mutants (Mut3), and a combination of mutations expected to be on the same haplotype as T1435A (Mut4). Other mutations that arose above 15% were introduced individually (Mut5 and Mut7), as well as combinations of those synonymous mutations potentially associated with the same haplotypes (Mut6 and Mut8). The two synonymous mutations likely associated with the T1435A haplotype were also introduced in combination (Mut9). Recombinant viruses were recovered and passaged once on Vero cells to generate working stocks. All viruses grew to high titers (~10^6-7^ PFU/mL) and had similar plaque morphologies (Fig. 4B and C).

### Evaluation of recombinant virus’s ability to infect *Culex* cells and mosquitoes.

**FIG 5:**
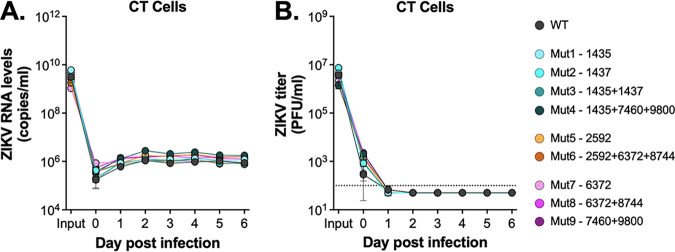
ZIKV mutants do not infect CT cells. *Cx. tarsalis* CT cells were infected at an MOI of 5 with ZIKV recombinant mutants, supernatant collected daily, and analyzed for (A) viral RNA via qRT-PCR and (B) infectious virus via plaque assays. Experiment was performed in biological triplicate (mean ± standard deviation). Dashed line represents limit of detection.

CT cells were infected with WT ZIKV and the nine recombinant viruses at an MOI of 5, and supernatant was sampled daily and evaluated for viral RNA and infectious virus (Fig. 5). We saw no indication of enhanced viral replication across any of the chimeric recombinant viruses compared to WT parental virus, revealing that none of the variants lead to increased replication in CT cells (Fig. 5A and B). We then infected two species of *Culex* mosquitoes (*Cx. tarsalis* and *Cx. quinquefasciatus*) with each of the viruses via a bloodmeal and looked at whole body positivity at 7 days postinfection (Table 4). There were only two positive mosquitoes in total, both in *Cx. tarsalis*; however, none of the recombinant viruses showed substantial improved infection in either species relative to the WT ZIKV (Table 4).

**TABLE 4:** Vector competence of *Culex* mosquitoes with ZIKV recombinant viruses^*a*^

Species	WT	Mut1	Mut2	Mut3	Mut4	Mut5	Mut6	Mut7	Mut8	Mut9
*Cx. tarsalis*	0/40 (0%)	1/40 (2.5%)	0/40 (0%)	0/40 (0%)	0/40 (0%)	1/40 (2.5%)	0/40 (0%)	0/40 (0%)	0/40 (0%)	0/40 (0%)
*Cx. quinquefasciatus*	0/38 (0%)	0/40 (0%)	0/40 (0%)	0/32 (0%)	0/40 (0%)	0/40 (0%)	0/25 (0%)	0/40 (0%)	0/40 (0%)	0/40 (0%)

a ZIKV infection was determined by standard plaque assay on Vero cells using whole body samples at day 7 postinfection.

### Passaging virus increased Aag2 infection.

**FIG 6:**
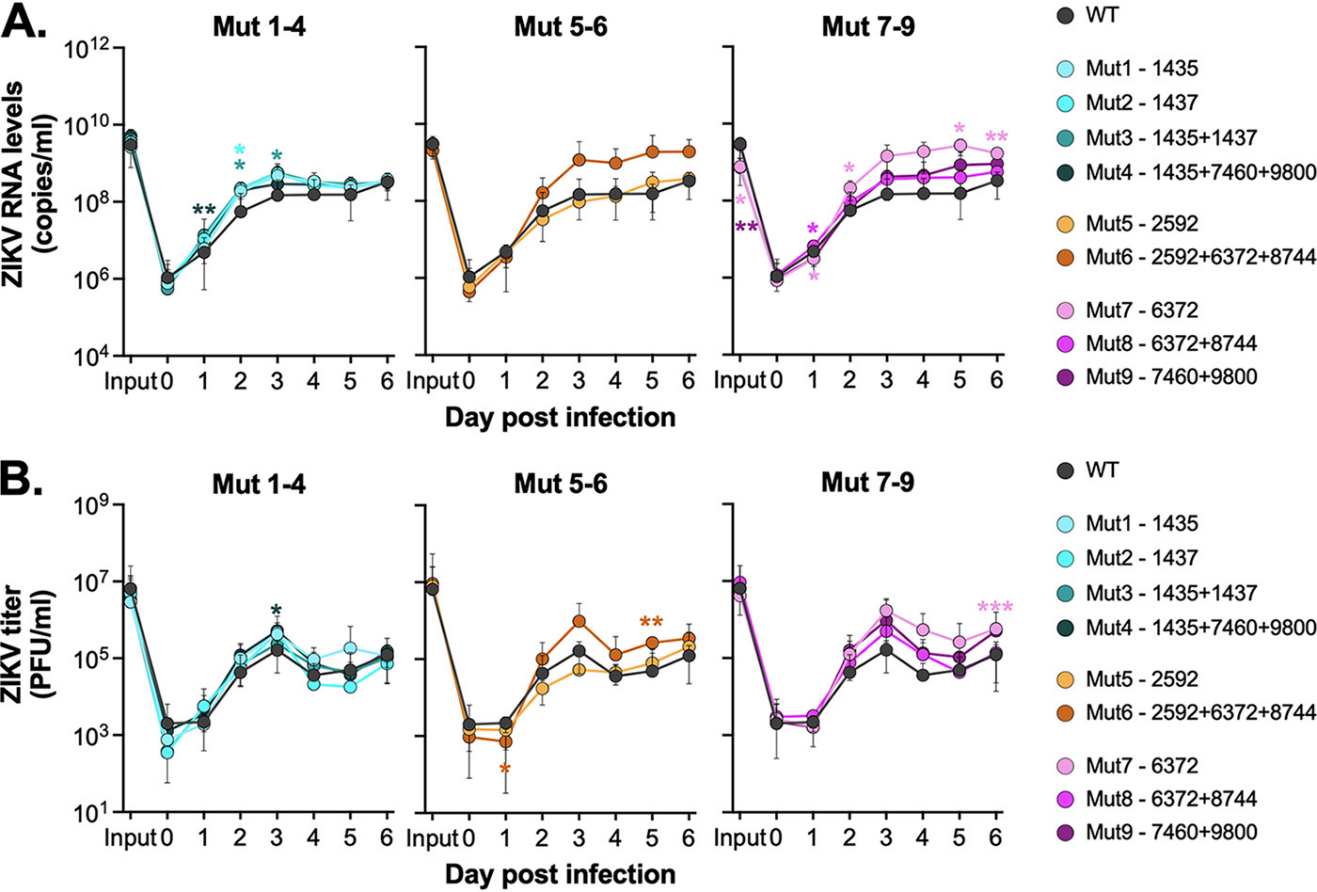
Increased replication of ZIKV recombinant viruses in Aag2 cells. *Ae. aegypti*-derived Aag2 cells were infected at an MOI of 5 with ZIKV recombinant viruses, supernatant collected daily, and analyzed for (A) viral RNA via qRT-PCR and (B) infectious virus via plaque assays. Recombinant viruses are grouped by the general location of their SNVs. Experiment was performed in biological triplicate (mean ± 95% confidence intervals). Two-way ANOVA with Dunnett’s multiple comparison was performed comparing each recombinant virus to WT at each time point. Only statistically significant relationships are shown (*, *P* < 0.05; **, *P* < 0.01; ***, *P* < 0.005).

Because we did not see evidence that passaging virus increased infection of *Culex* cells or mosquitoes, we hypothesized that passaging instead adapted viruses to Aag2 cells in culture. To test this, we evaluated all viruses for their ability to replicate in Aag2 cells at a high MOI (Fig. 6). We saw significant increases (*P* < 0.05) of viral RNA replication in many of the recombinants above that of WT ZIKV (Fig. 6A), suggesting adaptation to Aag2 cells. Replication of infectious virus was also significantly increased above that of WT ZIKV for some of the recombinant viruses (Fig. 6B).

**FIG 7:**
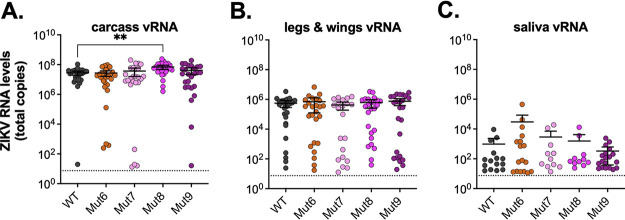
ZIKV recombinant viruses in *Ae. aegypti* mosquitoes. *Ae. aegypti* mosquitoes were infected with ZIKV recombinant viruses, and at day 7 salivated, dissected, and (A) carcass, (B) legs and wings, and (C) saliva were collected. Levels of viral RNA in mosquito tissues and saliva were quantified by qRT-PCR (mean ± 95% confidence intervals). Dashed line represents the limit of detection. Infection was performed in groups of 30 mosquitoes, only samples with detectable levels of virus are shown. One-way ANOVA with Dunnett’s multiple comparison was performed comparing each recombinant virus to WT. Only statistically significant relationships are shown (**, *P* < 0.005). Dashed line represents limit of detection.

We next sought to determine whether the increased Aag2 replication was general cell-culture adaptation, or adaptation to *Aedes* cells. We infected *Ae. aegypti* mosquitoes with four of the recombinant viruses (Mut 6–9) that had the highest replication in Aag2 cells (Fig. 6A and B) and compared that to WT ZIKV infection. At day 7 postinfection, mosquitoes were salivated and dissected to determine infection, dissemination, and transmission rates (Table 5). We saw similar infection rates across all viruses; however, dissemination and transmission rates were significantly decreased in some of the mutants relative to WT ZIKV (Fig. 6C). When comparing levels of viral RNA (vRNA) measured by qRT-PCR in each of these samples (carcass, legs and wings, and saliva) we saw similar levels of vRNA across viruses; however, Mut8 had significantly (*P* < 0.005) more vRNA compared to WT in the carcass only (Fig. 7A to C).

**TABLE 5:** Vector competence of *Ae. aegypti* mosquitoes with ZIKV recombinant viruses^*a*^

Tissue	WT	Mut6	Mut7	Mut8	Mut9
Carcass	26/30 (87%)	25/30 (83%)	24/30 (80%)	27/30 (90%)	23/30 (77%)
Legs and wings	24/30 (80%)	18/30 (60%)	13/30 (43%)^*c*^	18/30 (60%)	16/30 (53%)^*b*^
Saliva	7/30 (23%)	6/30 (20%)	3/30 (10%)	2/30 (7%)	0/30 (0%)^*c*^

a ZIKV infection was determined by standard plaque assay on Vero cells. Significant differences between Mut6-9 compared to WT for each tissue type using a Chi-square test are shown.

b *P* < 0.05.

c *P* < 0.005.

## DISCUSSION

In the present study, we attempted to adapt ZIKV to *Cx. tarsalis* cells to identify viral determinants of species specificity and blocks to efficient *Culex* infection and transmission. We found that in serial passaging of ZIKV on a mixed culture of *Ae. aegypti* and *Cx. tarsalis* cells with ever increasing proportions of *Cx. tarsalis* cells, the virus acquired numerous genetic changes at a consensus and minority level. When SNVs of interest were introduced into a ZIKV infectious clone, individually and in combination, we found that none resulted in increased infection of *Culex* cells or mosquitoes. Conversely, some of the mutations appeared to result in increased infection of Aag2 cells, but no increase in the infection, dissemination, or transmission of *Ae. aegypti* mosquitoes, suggesting overall adaptation to these cells and cell culture more generally.

Serial passage of viruses to adapt to cells, receptors, escape antibodies, etc. is a common technique used to identify viral genetic determinants. Serial passaging of ZIKV on *Culex* cells would be impossible due to the extremely low viral titers produced by *Culex* cells (Fig. 1). Coculturing two different cell types is less commonly used because it requires the cells to use the same, or compatible growth conditions (media type, temperature, etc.); however, the technique is becoming more common, especially within the fields of synthetic biology, drug development, and developmental biology (29, 30). Coculturing cells in the context of infection is occasionally described (31–33), but rarely with the goal of adapting a virus from a more permissive cell line to a less permissive one. We designed our approach loosely based on Morley et al. (25) which serially passaged SINV on a coculture of BHK-21 (highly permissive) and CHO (poorly permissive) cells. The technique of passaging virus on cells generally relies on the virus having some baseline infection in the cells of interest. We hypothesized that constant exposure of CT cells with ZIKV generated by Aag2 cells would ultimately allow for virus adaptation and increased replication in CT cells. However, in our experiments, the low level ZIKV replication in CT cells was likely not sufficient to allow adaptation and selection for improved infection in those cells. Since we observed the highest levels of prolonged replication with ZIKV strain 41525 *in vivo*, future strategies to evaluate *Culex*-adaptive SNVs may include passaging of ZIKV 41525 via microinjections *in vivo*. Additionally, it is possible that under different conditions of cell and mosquito infection (temperature, humidity, length of infection, etc.) that we would see higher permissiveness to infection.

Interestingly, a subset of the variants that emerged during coculture passaging (as CT cell ratios increased) resulted in improved viral replication in Aag2 cells; however, there was no increase in infection, dissemination, transmission rates in *Ae. aegypti* mosquitoes. These results reveal that while the virus adapted to mosquito cells in culture, the variants were likely more associated with tissue culture adaptation broadly, similar to what has been seen in other studies (34). Of note, Mut2 and Mut3 result in a loss of *N*-glycosylation at position 154 in E, without impact on virus titers or plaque formation in Vero cells and slightly increased replication in Aag2 cells. While *N*-glycosylation of E is important for flavivirus attachment to host cell receptors and viral release, as reviewed by Carbaugh and Lazear (35), a ZIKV N154Q mutant was previously shown to increase infectivity in C6/36 cells but reduce infection *in vivo* in mosquitoes and mice (36). We found no previous reports of the K590Q mutation in the helicase domain of NS3 (Mut7), which increased ZIKV replication in Aag2. However, since no significant increase in infectivity was observed *in vivo*, no follow-up studies were performed. It is currently still unclear whether the block to ZIKV infection observed in most *Culex* mosquitoes is efficient virus entry, lacking replication host factors, inability to evade immune responses, or virus maturation/egress. It is likely a combination of factors that overall suppress ZIKV replication in *Culex* cells to such an extent that adaptation is not possible in this type of a passaging set-up.

Importantly, our results reveal the challenge of arboviruses overcoming mosquito species barriers. In many examples with other arboviruses (WNV, CHIKV, MAYV, etc.), the viruses acquired genetic changes that improved viral infection or replication within an already competent mosquito species (20–24) but did not allow it to replicate in a previously noncompetent species. *Aedes* and *Culex* mosquitoes are genetically quite different (37), so ZIKV (and other *Aedes*-specific arboviruses) likely needs to acquire multiple genetic changes (likely in addition to other complex environmental factors) to allow the virus to gain the ability to infect *Culex* mosquitoes or other new species (38–40).

The 2015 to 2016 ZIKV outbreak was unprecedented, resulting in an estimated ~1 million human infections in the Americas alone, which is likely still a vast underreporting (41). As the virus spread across the globe between mosquitoes and humans from 2013 to 2017, it acquired many genetic changes, with some of these mutations becoming fixed in the American lineage viruses (prM-17, NS1-188, etc.) (42, 43). While the roles of these mutations are not fully elucidated, none have been shown to improve infection in *Culex* cells or mosquitoes. It is still possible that ZIKV could naturally acquire mutations and subpopulations of *Culex*-infecting viruses may exist in nature. It is also possible that complex environmental factors such as the mosquito microbiome or the presence of other *Culex*-borne pathogens (e.g., filarial worms) may influence ZIKV transmission in natural settings. However, it is unlikely that these mosquitoes are playing a major role in ZIKV transmission.

## MATERIALS AND METHODS

### Cells and viruses.

African green monkey-derived Vero cells were maintained in DMEM (Corning, number 10-017-CV) supplemented with 5% fetal bovine serum (FBS) at 37°C and 5% CO_2_. *Ae. aegypti*-derived Aag2 cells (44), and *Cx. tarsalis*-derived CT cells (45) were maintained in Schneider's media (Gibco, number 21720-024) supplemented with 7% FBS at 28°C. All media were further supplemented with 10 units/mL penicillin, 10 μg/mL streptomycin, and 2.5 μg/mL amphotericin B.

ZIKV strains PRVABC59 (Accession number KU501215) and MR766 (Accession number AY632535) were obtained from the CDC (Fort Collins Branch), while ZIKV strain 41525 (Accession number KU955591) was obtained from the University of Texas Medical Branch. These strains represent the American/Asian line (ZIKV PRVABC59), the East African line (MR766) and the West African line (41525). Passage histories have been previously described in detail (46). All wild-type/isolates of ZIKV were propagated in Vero cells by infection of a single flask of cells per virus at an MOI of 0.01, clarified by centrifugation, aliquoted, and stored at –80°C. ZIKV PRVABC59 infectious clones (wild-type and mutants) were propagated on Vero cells as previously described (28).

### Virus passaging.

Aag2 and CT cells were plated 1 day prior to infection at the noted proportions (from 90:10 to 10:90). For the initial infection, cells were infected with WT ZIKV PRVABC59 at a multiplicity of infection of 1 in three independent replicates. Virus was passaged on each proportion two consecutive passages (e.g., 90:10 two times, 80:20 two times, etc.) to allow the virus time to gradually adjust to the higher proportion of CT cells. The final passage contained 10% Aag2 cells and 90% CT cells due to concerns that we see a drastic drop of infectious virus titer in a 100% CT cells culture. Six days after infection, 500 μL of supernatant was transferred to the next passage of cells (for each replicate) while another 500 μL of supernatant were clarified by centrifugation and frozen at –80°C. This process was repeated for 18 passages total. The final cocultured virus was then passaged once on Vero cells to generate a working stock which was used for all experiments (denoted Co.18.1V, Co.18.2V and Co.18.3V).

### Growth curves.

CT or Aag2 cells were seeded one or 2 days prior to infection to be ~80% confluent at the time of infection. In biological triplicate for each virus, cells were infected for 1 h at 28°C, inoculum removed, cells washed twice with media, before fresh medium was added. Each day, 100 μL of supernatant (10% of volume) was sampled, immediately frozen at –80°C, and replaced with fresh media. Viral genomic RNA copies were determined as described below and viral titers were measured using standard plaque assay on Vero cells.

### qRT-PCR and plaque assays.

Viral RNA was isolated using Mag-Bind Viral DNA/RNA 96 kit (Omega Bio-tek) on a KingFisher Flex Magnetic Particle Processor (Thermo Fisher Scientific). qRT-PCR was performed using EXPRESS qPCR Supermix and Enzyme (Invitrogen), with previously published primers and probes (47) and an RNA-based ZIKV standard curve (48). For plaque assays, Vero cells were seeded 1 day prior to infection. Virus samples were serially diluted in DMEM supplemented with 1 to 2% FBS, added to Vero cell monolayer, and incubated for 1 h at 37°C. Cells were overlaid with 0.6% tragacanth media, incubated for 4 days, then fixed and stained with 20% ethanol and 0.1% crystal violet (Fisher Chemical, C481-100) in water. Plaques were counted manually.

### Mosquitoes.

Laboratory colonies of *Ae. aegypti* (established from wild populations collected in Poza Rica, Mexico in 2012), *Cx. quinquefasciatus* (established from wild populations collected in 1988 in Sebring County, Florida), *Cx. tarsalis* (established from a colony maintained by WK Reisen collected in 1953 from California), and *Cx. pipiens* (established from egg rafts collected in 2002 in Pennsylvania), were maintained at 28°C (*Ae. aegypti*) or 26 to 27°C (*Culex* spp.) with a 12:12 light:dark cycle (*Ae. aegypti*) or 16:8 light:dark cycle (*Culex* spp.) and 70%–80% relative humidity, with water and sugar provided *ad libitum*. Larvae were raised on powdered fish food (TetraMin, Tropic Flakes).

### Mosquito infections.

In a BSL-3/ACL-3 insectary, a single carton (per each virus) of female mosquitoes at 5 to 7 days post-eclosion were fed an infectious bloodmeal containing equal parts defibrinated calf blood (Colorado Serum Company, number 31033) and virus stock (~3–6 × 10^6^ PFU/mL final concentration). Bloodmeals were added to water-jacketed glass feeders sealed with a layer of hog’s gut and heated to 37°C via a water bath. Mosquitoes were allowed to feed for approximately 1 h, then anesthetized at 4°C and sorted for engorged females. *Culex* mosquitoes exposed to ZIKV by bloodmeal were held for 7 days and whole bodies were placed into microcentrifuge tubes containing mosquito diluent (PBS, 20% FBS, 50 μg/mL penicillin/streptomycin, 50 μg/mL gentamicin, and 2.5 μg/mL amphotericin B) and a steel bead, then homogenized. For intrathoracic microinjections, 5 to 7 day old mosquitoes were anesthetized at 4°C and held on ice. Female mosquitoes were intrathoracically injected with 138 nL of virus (4140 PFU/mosquito) using a Nanoject II microinjector and placed into a humid cage. Following blood meal or injection, mosquitoes (~30–60 per virus and infection condition, actual number is noted in tables) were held for 7 days with water and sugar provided *ad libitum* in a single carton per virus. Mosquitoes were cold-anesthetized, legs and wings removed, salivated into capillary tubes containing immersion oil (Cargille, type B high-viscosity) for 30 min, then carcasses were collected. Carcasses, and legs and wings were placed in tubes containing mosquito diluent, and a steel bead, then homogenized. Capillary tubes containing saliva were placed in tubes containing mosquito diluent, and centrifuged at 15,000 × *g* for 5 min at 4°C. For select experiments, complete mosquitoes were collected to only test infection rates. These mosquito bodies were treated in an identical fashion to carcasses, and legs and wings. All samples were stored at −80°C until sample processing. Detailed metadata for all mosquito infection experiments is provided in Table S1.

10.1128/msphere.00015-23.1 TABLE S1 Metadata of mosquito virus experiments. Download Table S1, XLSX file, 0.02 MB. Copyright © 2023 Gallichotte et al. 2023 Gallichotte et al. https://creativecommons.org/licenses/by/4.0/ This content is distributed under the terms of the Creative Commons Attribution 4.0 International license.

### Next-generation-sequencing (NGS).

Sequencing libraries were generated with an in-house optimized protocol for total viral RNA sequencing using Nextera XT (Illumina). Viral RNA was reverse transcribed into cDNA using SuperScript IV (Invitrogen) and random pentadecamers. RNA was then digested using RNaseH (New England Biolabs, number M02976), denatured, and random pentadecamers were annealed. NEBNext Ultra II Q5 Master Mix was added and samples incubated for 10 min at 72°C to complete second strand-synthesis. Ampure XP beads were used for DNA purification and Nextera XT (Illumina) was used for tagmentation (fragmentation and adapter addition) according to the manufacturer’s protocol. Libraries were amplified with indexing primers for 12 cycles using the NEBNext Ultra II Q5 Master Mix, size selected using Ampure XP beads, and real-time amplified using the KAPA HiFi HotStart Real-Time Library Amp kit (Roche). Library QC was performed, libraries were pooled at equimolar ratios, and sequenced on an Illumina NextSeq 500.

### Data analysis.

All NGS data were analyzed using a previously described workflow (49); this workflow was generated using Snakemake (50) and the workflow and related documentation can be found at https://bitbucket.org/murrieta/snakemake/src. Briefly, Read 1 and Read 2.fastq files from paired-end Illumina NextSeq 500 data were trimmed for Illumina adaptors and quality of phred scores < 30 from the 3′ and 5′ read ends using Cutadapt (51). Reads were then mapped to the ZIKV-PRVABC59 reference sequence (GenBank number KU501215) using MOSAIK (52). We used Picard (https://broadinstitute.github.io/picard/), Genome Analysis Toolkit (GATK) (53), and SAMtools (54) for variant calling preprocessing. To identify SNV’s as well as any inserts and deletions (indelS) we used LoFreq (55) with a cutoff of 1% variant frequency. Consensus sequences were generated using VCFtools (56).

### Cloning of recombinant viruses.

Recombinant ZIKVs containing SNVs of interest were introduced into our two-plasmid infectious ZIKV clone (28) using site-directed mutagenesis, Gibson assembly, and standard plasmid preparation. All recombinant viruses were confirmed by Sanger sequencing and virus was propagated from wild-type and mutant clones as previously described (28). Recombinant and wild-type viruses were passaged once on Vero cells to generate a working stock which was used in all experiments.

### Statistics.

Chi-square was used to compare infection rates across mosquito species, virus strains, infection types, and tissue samples. One-way and two-way ANOVAs with Dunnett’s multiple-comparison tests were used when comparing mutant virus levels to WT ZIKV. Details of specific statistical tests are noted in figure legends.

### Data availability.

All sequencing data are available through the NCBI SRA database (BioProject PRJNA933633).
